# Sigmoid Diverticulitis and Perforation Secondary to Biliary Stent Migration

**DOI:** 10.1155/2019/2549170

**Published:** 2019-05-19

**Authors:** Margaret Riccardi, Kaitlin Deters, Furrukh Jabbar

**Affiliations:** Department of General Surgery, Henry Ford Wyandotte Hospital, Wyandotte, MI, USA

## Abstract

**Introduction:**

Biliary stent migration occurs in 5-10% of patients. Generally, this is a benign process and stents pass or are retrieved endoscopically. In rare instances, intestinal perforation has occurred.

**Presentation of Case:**

A 79-year-old female presented with a one-day history of abdominal pain. She had undergone an ERCP four weeks previously for primary choledocholithiasis during which time a sphincterotomy and sphincteroplasty were performed, and stents were placed in the common bile duct. CT scan of the abdomen and pelvis demonstrated a biliary stent that had migrated into the sigmoid colon, appearing to perforate the colon with free air throughout the abdomen. Patient was taken for diagnostic laparoscopy and noted to have biliary stent perforating the sigmoid colon. Procedure was converted to open, and Hartmann's procedure was performed with end colostomy.

**Conclusion:**

Generally, biliary stent migration is a benign process, but in rare instances, intestinal perforation has occurred. Sites of perforation include the duodenum, distal small bowel, and colon. Perforation is more common with an additional pathology present such as hernias or diverticular disease. Migration and perforation also appear more common with straight biliary stents. In patients with known diverticular disease and straight biliary stents, considerations should be made for early stent removal.

## 1. Introduction

Endoscopic placement of plastic biliary stents for benign biliary disease has become a common procedure. Removal of stent is subsequently performed in six weeks to three months based on pathology and physician preference. Biliary stent migration occurs in 5-10% of patients. Generally, this is a benign process and stents pass without incident or are retrieved endoscopically [1]. In rare instances, intestinal perforation has occurred.

## 2. Presentation of Case

A 79-year-old female presented to the ED with a one-day history of severe left lower quadrant abdominal pain associated with chills and nausea. She had undergone an ERCP four weeks previously for primary choledocholithiasis during which time a sphincterotomy and sphincteroplasty were performed, and a 10 Fr stent with internal and external flaps and a 7 Fr stent with internal and external pigtails were placed in the common bile duct. On physical exam, the patient was tender to palpation in the left lower quadrant with voluntary guarding.

The patient was hypertensive on arrival to the ED, but all other vitals were within normal limits. A complete blood count, basic metabolic panel, liver profile, coagulation profile, and urinalysis were all within normal limits as well.

**Figure 1:**
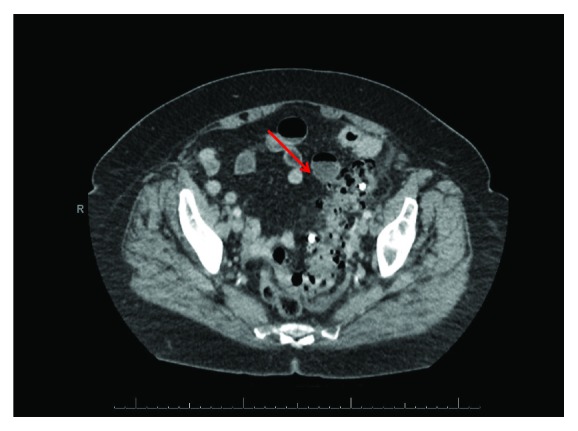
CT scan of biliary stent in sigmoid colon with diverticulosis and free air.

**Figure 2:**
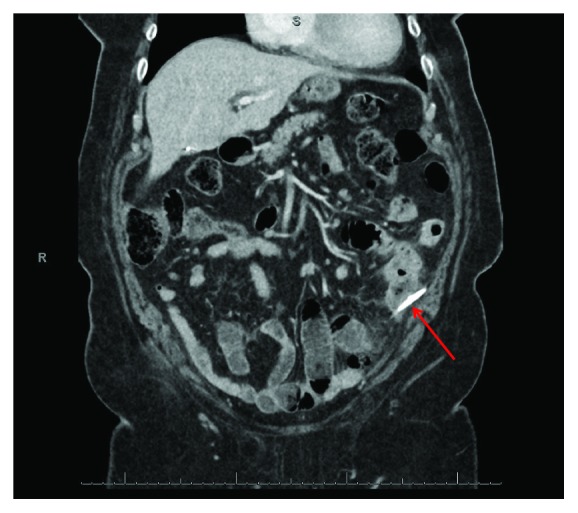
CT scan of biliary stent perforating sigmoid colon.

CT scan of the abdomen and pelvis demonstrated a biliary stent that had migrated into the sigmoid colon, appearing to penetrate the colon and possibly an adjacent loop of the small bowel. There was also free air and fluid throughout the abdomen (Figures 1 and 2).

**Figure 3:**
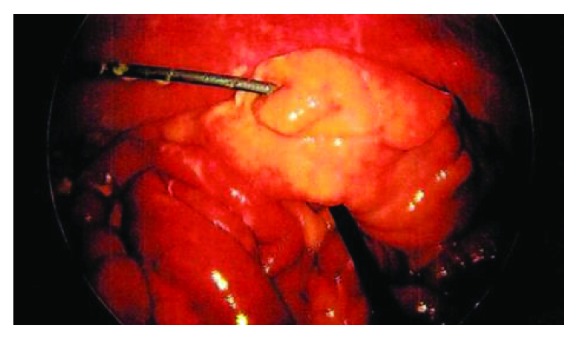
Laparoscopic image of biliary stent perforating sigmoid colon.

Patient was consented for surgery and taken to the operating room where a diagnostic laparoscopy was performed which visualized the biliary stent protruding from the sigmoid colon through a diverticulum (Figure 3). The procedure was converted to open, and Hartmann's procedure was performed with end colostomy. The patient sustained an NSTEMI perioperatively and required close monitoring but recovered well and was transferred to an inpatient rehabilitation facility on postoperative day 9.

## 3. Pathology

**Figure 4:**
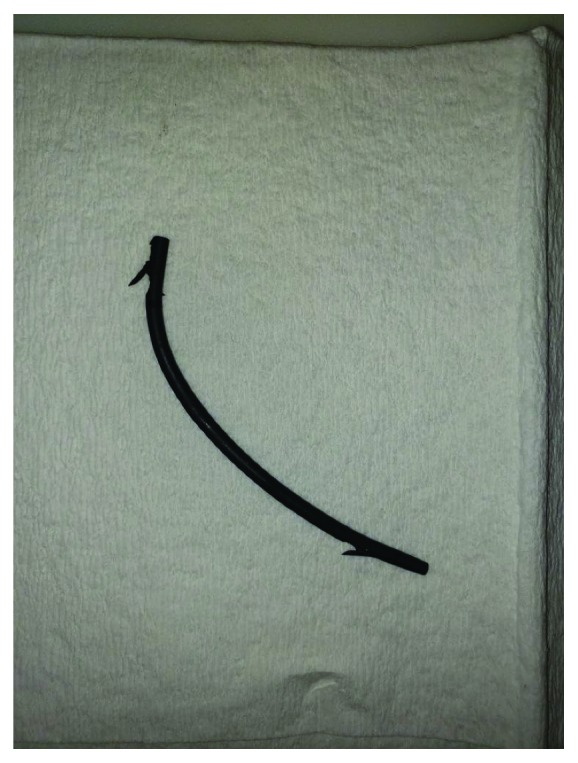
Gross pathologic images of biliary stent.

On gross examination of the sigmoid colon, the resected segment was 3.5 cm in length with moderate amount of adherent exudate, multiple outpouchings of the mucosa, and a perforation of 0.8 cm from the nearest end margin. The biliary stent was identified as a 10 × 0.3 cm segment of tan-brown rubbery tubing (Figure 4). The final pathological diagnosis was sigmoid diverticulosis and diverticulitis with perforation and acute serositis.

## 4. Discussion

While it is generally a benign process, biliary stent migration occurs in 5-10% of patients [1]. In rare instances, intestinal perforation has occurred. A review of the literature shows only twenty-five cases of intestinal perforation secondary to biliary stent migration. Sites of perforation include the duodenum, distal small bowel, and colon [2–8]. Perforation appears to be more common in patients with straight plastic stents, with soft pigtail stents rarely causing complications [2]. Perforation also appears to be more common in patients with other pathology such as diverticular disease or hernia [9]. This is also consistent with prior research suggesting colon perforation from foreign bodies is more common in patients with diverticular disease [10]. Given this increased risk of perforation with diverticula, consideration should be made for early stent removal in patients with known diverticular disease, particularly with the use of straight plastic biliary stents. Additionally, when considering stent placement, endoscopists should consider the placement of soft plastic stents with pigtails rather than straight plastic stents in patients with known diverticular disease.
